# Sparse bayesian learning for genomic selection in yeast

**DOI:** 10.3389/fbinf.2022.960889

**Published:** 2022-08-31

**Authors:** Maryam Ayat, Mike Domaratzki

**Affiliations:** ^1^ Lactanet, Sainte-Anne-deBellevue, QC, Canada; ^2^ Department of Computer Science, University of Western Ontario, London, ON, Canada

**Keywords:** genomic selection, bayesian learning, relevance vector machine, machine learning, kernel learning, marker assisted selection, explainable artificial intelligence

## Abstract

Genomic selection, which predicts phenotypes such as yield and drought resistance in crops from high-density markers positioned throughout the genome of the varieties, is moving towards machine learning techniques to make predictions on complex traits that are controlled by several genes. In this paper, we consider sparse Bayesian learning and ensemble learning as a technique for genomic selection and ranking markers based on their relevance to a trait. We define and explore two different forms of the sparse Bayesian learning for predicting phenotypes and identifying the most influential markers of a trait, respectively. We apply our methods on a *Saccharomyces cerevisiae* dataset, and analyse our results with respect to existing related works, trait heritability, as well as the accuracies obtained from linear and Gaussian kernel functions. We find that sparse Bayesian methods are not only competitive with other machine learning methods in predicting yeast growth in different environments, but are also capable of identifying the most important markers, including both positive and negative effects on the growth, from which biologists can get insight. This attribute can make our proposed ensemble of sparse Bayesian learners favourable in ranking markers based on their relevance to a trait.

## 1 Introduction

Genomic Selection (GS) is a computational and statistical problem which predicts phenotypes such as growth and fertility in livestock (Kemper and Goddard, 2012; García-Ruiz et al., 2016), and yield and drought resistance in crops (Beyene et al., 2015), using genetic information of individuals, that is, sequences of genome-wide molecular markers. Single Nucleotide Polymorphisms (SNPs) are the most common type of genetic markers. GS is ideal for complex traits, which are controlled by many genes with different effects across the genome (Poland and Rutkoski, 2016). GS in plants or animals are mainly used in the breeding industry to facilitate the selection of superior genotypes and accelerate the breeding cycle (Meuwissen et al., 2016; Crossa et al., 2017).

Previous work on GS has focused primarily on statistical models, including Best Linear Unbiased Prediction (BLUP) and its variants (Bloom et al., 2013; Habier et al., 2013; Spindel et al., 2016; You et al., 2016; Zhang et al., 2016). However, machine learning methods, such as random forests (Breiman, 2001) and Support Vector Machines (SVMs) (Schölkopf et al., 2003), have also seen an increasing interest in GS research on plants (Jannink et al., 2010; Okser et al., 2014; Blondel et al., 2015; Li et al., 2015; Grinberg et al., 2018, 2016) and animals (Moser et al., 2009; González-Recio et al., 2014; Yao et al., 2016). In this research, we use sparse Bayesian learning (Tipping, 2001) for predicting phenotypes and identifying influential markers on growth in the yeast *Saccharomyces cerevisiae*. This learning method uses Bayesian inference to obtain sparse solutions for regression and classification tasks. The model is also known as the Relevance Vector Machine (RVM), as it can be viewed as a kernel-based model of identical form to the SVM, which is a theoretically well-motivated classification algorithm in modern machine learning (Mohri et al., 2012). Although the predictive performance of RVMs is similar to SVMs, they have some advantages that SVMs lack, such as having probabilistic outputs and the ability to work with arbitrary kernel functions. More importantly for our purposes, RVMs construct much sparser models based on identifying more meaningful representatives of training data compared to the SVMs (Tipping, 2001). We use these representatives to help link phenotype predictions and identification of important markers in the yeast genome.

In this work, we consider the association problem as an embedded feature ranking problem wherein features are biological markers (e.g., SNPs), and the feature selection process is part of the predictive model construction. Then, the ranks of features based on their relevance to the trait will give candidate markers which can be further investigated. Motivated by the sparse solution property of sparse Bayesian learning, we investigate a novel ensemble architecture for feature selection and ranking. More precisely, we merge sparse Bayesian learning, ensemble and bagging techniques for ranking influential SNP markers on a quantitative trait. Note that there are also limited studies that used sparse Bayesian method for feature selection in bioinformatics (Li et al., 2002; Krishnapuram et al., 2004; Cawley and Talbot, 2006; Yang et al., 2017). However, this work, specifically on genes associated with disease, was only for classification, and did not incorporate ensemble techniques.

## 2 Data and methods

### 2.1 Dataset

Bloom et al. (2013) developed 1,008 haploid strains of *Saccharomyces cerevisiae* as a result of crosses between laboratory and wine strains of the yeast. The parent strains had sequence level differences of 0.5%. The genotypes consist of SNP markers that correspond to 11,623 sequence locations in the genome. The locations are coded as 1 if the sequence variation came from the wine strain parent, or 0 if it came from the laboratory strain parent.

Bloom et al. modified the environment of 1,008 yeast strains in 46 different conditions (first column in Table 1), and measured the population growth under those different conditions. For example, they varied the basic chemicals used for growth (e.g., galactose, maltose), or added minerals (e.g., copper, magnesium chloride), then measured growth in that condition. To quantify growth, Bloom et al. calculated the radius of the colonies from an image taken after approximately 48 h of growth. Some results, such as irregular colonies, were removed and treated as missing data. Most conditions had more than 90% of readings included.

**TABLE 1:** Coefficient of determination (*R* ^2^) and standard deviation of the best performing model (std) of RVM predictions among the 46 traits.

**Trait**	**Linear Basis**	**Linear**	**Gaussian**	**Best RVM**	**std**
Cadmium Chloride	0.639	0.033	0.454	Linear Basis	0.005
Caffeine	0.074	0.216	0.233	Gaussian (*γ*_3_)	0.006
Calcium Chloride	0.113	0.273	0.287	Gaussian (*γ*_3_)	0.007
Cisplatin	0.133	0.287	0.29	Gaussian (*γ*_3_)	0.005
Cobalt Chloride	0.258	0.439	0.466	Gaussian (*γ*_2_)	0.006
Congo red	0.327	0.467	0.491	Gaussian (*γ*_1_)	0.006
Copper	0.146	0.334	0.379	Gaussian (*γ*_3_)	0.01
Cycloheximide	0.317	0.473	0.514	Gaussian (*γ*_1_)	0.005
Diamide	0.277	0.473	0.483	Gaussian (*γ*_2_)	0.005
E6 Berbamine	0.211	0.375	0.414	Gaussian (*γ*_2_)	0.008
Ethanol	0.276	0.457	0.476	Gaussian (*γ*_2_)	0.006
Formamide	0.114	0.207	0.25	Gaussian (*γ*_2_)	0.006
Galactose	0.076	0.206	0.241	Gaussian (*γ*_3_)	0.008
Hydrogen peroxide	0.234	0.343	0.397	Gaussian (*γ*_2_)	0.01
Hydroquinone	0.087	0.139	0.208	Gaussian (*γ*_3_)	0.009
Hydroxyurea	0.12	0.296	0.342	Gaussian (*γ*_2_)	0.01
Indoleacetic Acid	0.128	0.255	0.313	Gaussian (*γ*_2_)	0.007
Lactate	0.36	0.542	0.555	Gaussian (*γ*_2_)	0.005
Lactose	0.374	0.553	0.574	Gaussian (*γ*_2_)	0.008
Lithium Chloride	0.531	0.597	0.678	Gaussian (*γ*_1_)	0.006
Magnesium Chloride	0.102	0.245	0.255	Gaussian (*γ*_3_)	0.005
Magnesium Sulfate	0.187	0.366	0.41	Gaussian (*γ*_3_)	0.005
Maltose	0.409	0.484	0.523	Gaussian (*γ*_2_)	0.005
Mannose	0.079	0.213	0.197	Linear	0.007
Menadione	0.216	0.389	0.411	Gaussian (*γ*_3_)	0.006
Neomycin	0.422	0.583	0.596	Gaussian (*γ*_2_)	0.003
Paraquat	0.31	0.442	0.454	Gaussian (*γ*_2_)	0.005
Raffinose	0.185	0.385	0.388	Gaussian (*γ*_3_)	0.007
SDS	0.199	0.36	0.398	Gaussian (*γ*_2_)	0.004
Sorbitol	0.176	0.343	0.364	Gaussian (*γ*_3_)	0.009
Trehalose	0.326	0.48	0.503	Gaussian (*γ*_2_)	0.005
Tunicamycin	0.417	0.594	0.622	Gaussian (*γ*_1_)	0.006
4-Hydroxybenzaldehyde	0.23	0.34	0.367	Gaussian (*γ*_2_)	0.008
4NQO	0.44	0.496	0.512	Gaussian (*γ*_2_)	0.005
5-Fluorocytosine	0.215	0.323	0.378	Gaussian (*γ*_2_)	0.008
5-Fluorouracil	0.326	0.505	0.559	Gaussian (*γ*_2_)	0.005
6-Azauracil	0.152	0.3	0.304	Gaussian (*γ*_3_)	0.005
Xylose	0.282	0.455	0.478	Gaussian (*γ*_3_)	0.004
YNB	0.379	0.224	0.515	Gaussian (*γ*_1_)	0.009
YNB:ph3	0.059	0.18	0.177	Gaussian (*γ*_3_)	0.005
YNB:ph8	0.203	0.327	0.361	Gaussian (*γ*_2_)	0.006
YPD	0.368	0.266	0.511	Gaussian (*γ*_1_)	0.008
YPD:15C	0.211	0.334	0.356	Gaussian (*γ*_2_)	0.006
YPD:37C	0.473	0.566	0.611	Gaussian (*γ*_2_)	0.006
YPD:4C	0.18	0.406	0.438	Gaussian (*γ*_2_)	0.005
Zeocin	0.316	0.46	0.475	Gaussian (*γ*_3_)	0.004

### 2.2 Sparse Bayesian learning

Sparse Bayesian modelling (Tipping, 2001; Tipping and Faul, 2003) is an approach for learning a prediction function *y* (**x**; **w**), which is expressed as a linear combination of basis functions:

$$y\left(x;w\right)=\overset{M}{\underset{m=1}{\sum }}w_{m}\rho_{m}\left(x\right)=w^{T}\rho \left(x\right),$$ (1)

where $\rho \left(x\right)={\left(\rho 1\left(x\right),\ldots ,\rho_{M}\left(x\right)\right)}^{T}$ are basis functions, generally non-linear, and *w*_1_, … , *w*_*M*_ are the adjustable parameters, called weights. Given a dataset of input-target training pairs ${\left\{\left(x_{i},t_{i}\right)\right\}}_{i=1}^{N}$ , the objective of the sparse Bayesian method is to estimate the target function *y* (**x**; **w**), while retaining as few basis functions as possible. The sparse Bayesian algorithm often generates exceedingly sparse solutions (i.e., few non-zero parameters *w*_*i*_).

In a particular specialization of (1), such as the one that SVMs use, *M* = *N* and the basis functions take the form of kernel functions, one for each data point **x**_*m*_ in the training set. In this case, we get *ρ*_*m*_(**x**) = *K* (**x**, **x**_*m*_), where K:X×X→R is the kernel function. This form of sparse Bayesian modelling is called the Relevance Vector Machine (RVM). Tipping, (2000) introduced the RVM method as an alternative to the SVM method of Vapnik, (1998). However unlike SVMs, where the kernel functions must be Positive Definite Symmetric (PDS) (Hofmann et al., 2008), we can use arbitrary basis sets in the RVM.

Assuming that the basis functions have the form of kernel functions, we illustrate the sparse Bayesian algorithm for regression as follows. Corresponding algorithms for arbitrary basis functions can be easily induced from them.

#### 2.2.1 *Relevance vector regression*

We follow the framework developed by Tipping, (2001). In the regression framework, the targets $t={\left(t_{1},\ldots ,t_{N}\right)}^{T}$ are real-valued labels. Each target *t*_*i*_ is representative of the true model *y*_*i*_, but with the addition of noise *ϵ*_*i*_: *t*_*i*_ = *y* (**x**_*i*_) + *ϵ*_*i*_, where $\epsilon_{i}∼N\left(0,\sigma^{2}\right)$ . This means *p* (*t*_*i*_∣**x**_*i*_, **w**, *σ* ^2^) = *N* (*y* (**x**_*i*_), *σ* ^2^), or

$$p\left(t|w,\sigma^{2}\right)={\left(2\pi \sigma^{2}\right)}^{-N/2}\exp\left\{-\frac{1}{2\sigma^{2}}\|t-\Phi w{\|}^{2}\right\},$$ (2)

where $w={\left(w_{1},\ldots ,w_{N}\right)}^{T}$ , and the data is hidden in the design matrix (kernel matrix) $P={\left[\rho \left(x_{1}\right),\ldots ,\rho \left(x_{N}\right)\right]}^{T}$ , wherein $\rho \left(x_{i}\right)={\left[K\left(x_{i},x_{1}\right),\ldots ,K\left(x_{i},x_{N}\right)\right]}^{T}$ . For clarity, we omit the implicit conditioning on the set of input vectors {**x**_*i*_} in (2) and subsequent expressions.

We infer weights using a fully probabilistic framework. Specifically, we define a Gaussian prior distribution with zero mean and ${\alpha_{i}}^{-1}$ variance over each *w*_*i*_: $p\left(w_{i}|\alpha_{i}\right)=N\left(0,{\alpha_{i}}^{-1}\right),$ or:

$$p\left(w|\alpha \right)=\overset{N}{\underset{i=1}{\prod }}N\left(0,{\alpha_{i}}^{-1}\right).$$ (3)

The sparsity of the RVM is a result of the independence of the hyperparameters $\alpha ={\left(\alpha_{1},\ldots ,\alpha_{N}\right)}^{T}$ one per basis function (i.e., weight), which moderate the strength of the prior information (Li et al., 2009).

Using Bayes’ rule and having the prior distribution and likelihood function (3) and (2), the posterior distribution over the weights would be a multivariate Gaussian distribution:

$$p\left(w|t,\alpha ,\sigma^{2}\right)=\frac{p\left(t|w,\sigma^{2}\right)p\left(w|\alpha \right)}{p\left(t|\alpha ,\sigma^{2}\right)}=N\left(\mu ,\Sigma \right),$$ (4)

where the covariance and the mean are:

$$\Sigma ={\left(\sigma^{-2}\Phi^{T}\Phi +A\right)}^{-1},$$ (5)

$$\mu =\sigma^{-2}\Sigma \Phi^{T}t,$$ (6)

and **A** = diag (*α*_1_, … , *α*_*N*_).

The likelihood distribution over the training target **t**, given by (2), is marginalized with respect to the weights to obtain the marginal likelihood for the hyperparameters:

$$p\left(t|\alpha ,\sigma^{2}\right)=\int p\left(t|w,\sigma^{2}\right)p\left(w|\alpha \right)dw=N\left(0,C\right),$$ (7)

where the covariance is given by *C* = *σ* ^2^ **I** + Φ**A** ^−1^Φ^*T*^. Values of ***α*** and *σ* ^2^ which maximize (7) cannot be obtained in closed form, thus the solution is derived via an iterative maximization of the marginal likelihood *p* (**t**∣ ***α***, *σ* ^2^) with respect to ***α*** and *σ* ^2^:

$${\alpha_{i}}^{\mathrm{new}}=\frac{1-\alpha_{i}\Sigma_{ii}}{{\mu_{i}}^{2}},$$ (8)

$${\left(\sigma^{2}\right)}^{\mathrm{new}}=\frac{\left\|t-\Phi \mu \right\|}{N-\sum_{i=1}^{N}\left(1-\alpha_{i}\Sigma_{ii}\right)}.$$ (9)

By iterating over (5), (6), (8), and (9), the RVM algorithm reduces the dimensionality of the problem when *α*_*i*_ tends to infinity (note that *α*_*i*_ has a negative power in (3)) (Ben-Shimon and Shmilovici, 2006). The algorithm stops when the likelihood *p* (**t**∣ ***α***, *σ* ^2^) stops increasing. The non-zero elements of **w** are called Relevance Values. The input vectors which correspond to the relevance values are called Relevance Vectors (RVs) as an analogy to Support Vectors in the SVM (Ben-Shimon and Shmilovici, 2006). Having the relevance vectors, ${\left\{x_{r}\right\}}_{r=1}^{\left|RVs\right|}$ , and the relevance values, ${\left\{w_{r}\right\}}_{r=1}^{\left|RVs\right|}$ , the RVM makes prediction on a new data instance x_*_:

$$y_{*}=\overset{\left|RVs\right|}{\underset{r=1}{\sum }}w_{r}K\left(x_{*},x_{r}\right),$$

where $\left|RVs\right|$ denotes the cardinality of the set of relevance vectors.

Given the kernel matrix, the computationally intensive part of the RVM algorithm is the matrix inversion in (6) which requires *O*(*N* ^3^) operations for a dataset of size *N*. This is similar to the *O*(*N* ^3^) training time for SVMs using standard algorithms.

The regression framework can be extended to the classification case using the approximation procedure presented in (Tipping, 2000).

### 2.3 Kernel RVM versus basis RVM

Kernel methods are flexible techniques that allow the user to select a kernel function that enables tools such as RVMs and SVMs to define non-linear decision boundaries (Cortes et al., 2004). For example, consider a binary classification problem in which input patterns are not linearly separable in the input space (i.e., inputs cannot be separated into two classes by passing a hyperplane between them). In such a case, one solution is to use a non-linear mapping of the inputs into some higher-dimensional feature space in which the patterns are linearly separable. Then, we solve the problem (i.e., finding the optimal hyperplane) in the feature space, and consequently, we will be able to identify the corresponding non-linear decision boundary for the input vectors in the input space. To do this procedure, a kernel method only requires a function K:X×X→R , which is called a kernel over the input space *X*. For any two input patterns **x**_*i*_, **x**_*j*_ ∈ *X*, *K* (**x**_*i*_, **x**_*j*_) is the dot product of vectors *φ*(**x**_*i*_) and *φ*(**x**_*j*_) for some mapping *φ*: *X* → *H* to a feature space *H*:

$$\forall x_{i},x_{j}\in X,\;K\left(x_{i},x_{j}\right)=\left\langle \varphi \left(x_{i}\right),\varphi \left(x_{j}\right)\right\rangle .$$

In this research, we define sparse Bayesian learning in such a way that we can discriminate between kernel and basis functions, i.e., “kernel” RVM versus “basis” RVM. The basis RVMs, which do not have counterparts in SVMs, will be mainly used to enable feature selection. For example, we define two types of linear RVMs, which we call linear kernel RVMs and linear basis RVMs. In a linear kernel RVM, the basis functions in (1) are linear kernel functions, i.e.,

$$\rho_{m}\left(x\right)=K\left(x,x_{m}\right)=\left\langle x,x_{m}\right\rangle .$$

When we use linear kernels, in fact we have no mapping. In other word, there is no feature space (as we use input vectors directly), so our estimator tries to pass a hyperplane through input vectors in the input space (e.g., in the case of regression).

In our linear basis RVM, the basis functions are linear and equal to the features of the input vectors, i.e.,

$$\rho_{m}\left(x\right)=x^{\left[m\right]},$$

where **x** ^[*m*]^ refers to the *m*th feature in an input vector **x** with *M* dimensions. We can view it as if we have no basis function in a linear basis RVM, as we use input vectors directly in (1) instead:

$$y\left(x;w\right)=w^{T}x.$$

That is, in this formulation with a linear basis RVM, the model is a Bayesian linear regression using the RVM optimization and anticipated sparsity. We can restate (2) with weights $w={\left(w_{0},w_{1},\ldots ,w_{M}\right)}^{T}$ , where *M* is the number of features, and the design matrix is

$$\Phi_{N\times \left(M+1\right)}=\left[\begin{array}{cccc} 1 & {x_{1}}^{\left[1\right]} & \; & {x_{1}}^{\left[M\right]}\\ 1 & {x_{2}}^{\left[1\right]} & \; & {x_{2}}^{\left[M\right]}\\ & & \cdots & \\ 1 & {x_{N}}^{\left[1\right]} & \; & {x_{N}}^{\left[M\right]} \end{array}\right],$$ (10)

where the first column handles the intercept *w*_0_, and *N* is the number of training individuals.

Thus, this linear basis RVM will find the RVs which corresponds to the features; i.e., the obtained sparsity will be in the feature set rather than the training individuals. This is exactly what we expect from a feature selection method. Therefore, this RVM can perform target prediction as well as feature selection. For example, in a GS in crop breeding, the individuals are breeds of a crop, the features are the markers (SNPs), and a phenotype is a target. Then, a linear basis RVM would identify a subset of relevant markers to that phenotype, while it is trained for phenotype prediction.

Similar to linear RVMs, we can define any other non-linear RVMs (i.e., Gaussian RVM as Gaussian kernel RVM or Gaussian basis RVM). In our experiments, we apply kernel RVMs with different PDS kernel types to investigate how they perform in predicting phenotypes. However, we only examine linear basis RVMs for phenotype prediction and influential marker identification.

Compared to the SVM method, we should note that there is not an SVM counterpart for a basis RVM, as the design matrix (10) resembles a non-PDS function which specifically cannot be used in an SVM. In a kernel RVM, we can use PDS kernels, such as polynomial and Gaussian kernels, or non-PDS kernels, such as sigmoid kernels (neural network kernels (Schölkopf and Smola, 2002)). In the case of using PDS kernels, the kernel RVM prediction accuracies will be comparable to the SVM results.

#### 2.3.1 *Kernel types*

In our experiments with kernel RVMs, we use Gaussian, polynomial and linear kernels. For any constant *γ* > 0, the Gaussian kernel is the kernel $K:R^{N}\to R$ :

$$\forall x,x^{\prime }\in R^{N},\;K\left(x,x^{\prime }\right)=\exp\left(-\gamma {\left\|x-x^{\prime }\right\|}^{2}\right),$$

where ‖**x**‖ is the norm of the vector **x**. Also, a polynomial kernel of degree *d* such as *K* is defined by:

$$\forall x,x^{\prime }\in R^{N},\;K\left(x,x^{\prime }\right)={\left(x\cdot x^{\prime }+c\right)}^{d},$$

for a fixed constant *c* ≥ 0. A linear kernel is a polynomial kernel with *c* = 0 and *d* = 1.

#### 2.3.2 *RVM as a phenotype predictor*

We consider the yeast dataset as 46 separate regression problems: we construct a separate RVM model for predicting growth under each of 46 conditions. We train each RVM with linear basis function, linear kernel, Gaussian kernel (with different values of *γ* parameter), and a set of *n*-gram kernels. Using the coefficient of determination (*R* ^2^) as measure, and running 10 times of 10-fold cross-validation (each time with random different folds), we evaluate the results of RVM models. As the process for this dataset along with repeating cross-validations is computationally heavy, the process is done in parallel on the WestGrid (www.westgrid.ca) platform. To facilitate direct comparison between our results and those of previous work below, we use *R* ^2^ as a measurement of the goodness of fit between the predicted and actual measurements on the yeast dataset (i.e., predicted versus actual colony size).

### 2.4 Ensemble RVM

In an ensemble, a set of classifiers is trained and for new predictions, the results of each of the classifiers is combined to obtain a final result (Dietterich, 2000). Ensembles often produce better predictive performance than a single model by decreasing variance (bagging), bias (boosting), or improving predictions (stacking) (Zhou, 2012). Moreover, ensemble techniques have the advantage of handling large data sets and high dimensionality because of their divide-and-conquer strategy. Random Forests (Breiman, 2001) and Gradient Boosting Machines (GBMs) (Friedman, 2001) are examples of ensemble methods.

In this research we use ensemble RVM with bagging approach. Bagging (bootstrap aggregating (Breiman, 1996)) is based on bootstrapping, where sample subsets of a fixed size are drawn with replacement from an initial set of samples. In bagging, a large number of separate classifiers in an ensemble are trained on separate bootstrap samples and their predictions are aggregated through majority voting or averaging. Bagging is commonly used as a resolution for the instability problem in estimators.

We use ensembles of basis RVMs for feature selection and ranking. Each RVM model in an ensemble finds a set of representatives (the RVs) which represent important features. Then, aggregating RVs of the ensemble lets rank the features. The top ranked markers are chosen based on a threshold. In other words, we define the most influential markers as those who are chosen by a specific percentage of the RVMs in the ensemble as RVs. Ranking mechanisms allow us to reduce dimensionality and enhance generalization (Saeys et al., 2007). Furthermore, they enable us to recognize interpretable or insightful features in the model.

We use SpareBayes software package for Matlab (Tipping, 2019) to implement the RVMs in this research. The code for the RVM experiments is available at https://github.com/maryamayat/YeastRVM.

## 3 Results and discussion

### 3.1 Predicting phenotypes

The prediction accuracies plus the standard deviation of cross-validation results in the best RVM model are shown in Table 1 (Note the following Gaussian parameters that are used in the table: *γ*_1_ = 1e − 4, *γ*_2_ = 2e − 4, and *γ*_3_ = 3e − 4.). The value reported for the *γ* parameter of Gaussian function in the table is the best of a range of values for model selection. Note that Gaussian kernel RVMs mostly produce promising results. Even in a trait such as Mannose, the linear kernel RVM shows a slightly better accuracy than the Gaussian. The only exception is Cadmium Chloride in which linear basis RVM presents a significantly better accuracy. The RVM models are stable, based on the standard deviations. In following subsections, we analyse the results with more details. In practice, optimal values for *γ* can be obtained through well-studied grid search techniques similar to those implemented for, e.g., SVM classification and regression. The Friedman and Nemenyi tests indicate that the mean of coefficients of determination for Gaussian RVMs are significantly different from Linear Basis and Linear RVM (*p* = 0.001 for both cases).

#### 3.1.1 *Linear kernel RVM* versus *linear basis RVM*

As explained before, a linear basis RVM can be viewed as an RVM with no basis function, as we use input vectors directly in the data model instead. Similarly when we use linear kernels, it means we do not map the inputs into a higher dimensional feature space, so our estimator tries to pass a hyperplane through input vectors in the input space. Here, we might expect that both linear kernel and linear basis RVMs produce similar results or with subtle difference, as both are linear and in the same space. However, that is not the case, i.e., linear kernel RVM and linear basis RVM produces different hyperplanes as we see in the results in Table 1. Consider Cadmium Chloride and YPD:4C, as two extreme examples. In the former, the linear basis RVM has high accuracy, while in the latter the linear kernel RVM shows higher accuracy. As a corollary we can say that linear basis RVM produces results which classic linear SVM is not able to. We know that the linear kernel cannot be more accurate than a properly tuned Gaussian kernel (Keerthi and Lin, 2003), but we cannot conclude the same for the linear basis function. Therefore, even if we have conducted a complete model selection using the Gaussian kernel RVM for a problem, it is still valuable to consider the linear basis RVM, just as we saw linear basis superiority to Gaussian kernel in Cadmium Chloride.

#### 3.1.2 *Heritability* versus *accuracies*

Bloom et al. (2013) provided estimates for narrow-sense and broad-sense heritability for the yeast dataset. They considered broad-sense heritability as the contribution of additive genetic factors (i.e., narrow-sense heritability) and gene-gene interactions. Thus, the broad-sense heritability is always greater than the narrow sense heritability, and their difference can be interpreted as a measurement of gene-gene interactions (Bloom et al., 2013). The broad-sense heritability estimates among the 46 traits ranged from 0.40 (YNB:ph3) to 0.96 (Cadmium Chloride), with a median of 0.77. Also, the narrow-sense heritability estimates ranged from 0.21 (YNB:ph3) to 0.84 (Cadmium Chloride), with a median of 0.52. Using the difference between two heritability measures, Bloom et al. estimated the fraction of genetic variance due to gene-gene interactions, which ranged from 0.02 (5-Fluorouracil) to 0.54 (Magnesium Sulfate), with a median of 0.30. Therefore, the genetic basis for variation in some traits, such as 5-Fluorouracil, is almost entirely due to additive effects, while for some others, such as Magnesium Sulfate, approximately half of the heritable component is due to gene-gene interactions.

**FIGURE 1:**
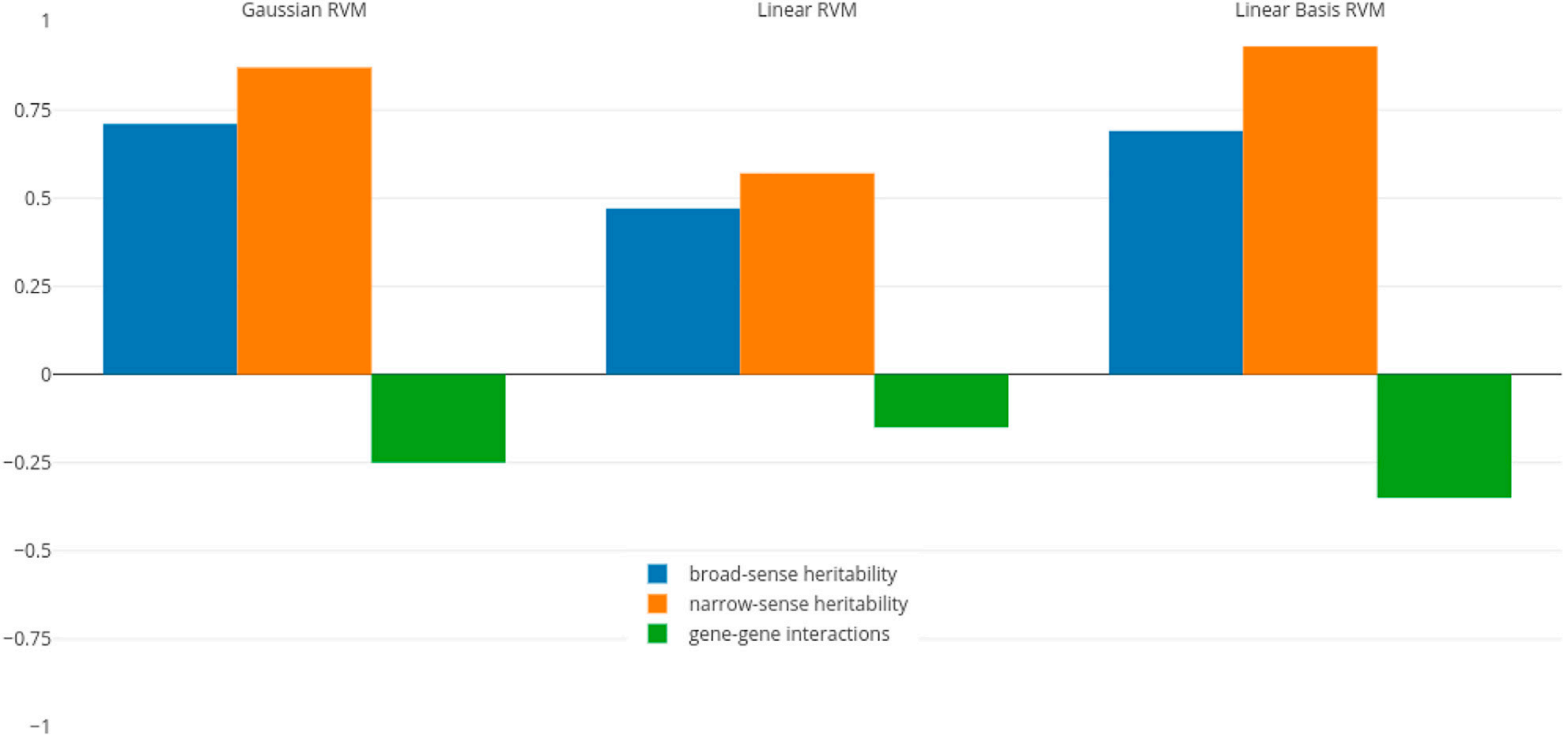
Pearson correlation coefficient between RVM accuracies and different heritability measures.

To determine if there is a correlation between heritability and RVM prediction accuracies, we calculated the Pearson correlation coefficient between estimates of heritability and prediction accuracies. The correlation coefficients in three RVM categories (Gaussian, linear, and linear basis) are shown in Figure 1.

The values related to the broad- and narrow-sense heritability (blue and orange bars) indicate that heritability and RVM accuracies, particularly in Gaussian and linear basis RVMs, have strong positive association. In other words, we will have better predictions when the amount of heritability increases. In particular, a higher narrow-sense heritability yields better prediction rates for the RVM predictor.

To determine if RVMs are less successful in predicting traits with larger non-additive effects, we also calculated the correlation coefficient between RVM accuracies and gene-gene interactions effects (green bars in the figure). These values indicate that gene-gene effects and accuracies, particularly in Gaussian and linear RVMs, have small negative association, indicating that we cannot infer the RVM performance is deteriorating when gene-gene interactions effects increases. This confirms previous results where non-parametric and semi-parametric machine learning techniques, such as SVMs, RKHS, and random forests, have been shown to have good prediction abilities for non-additive traits (Howard et al., 2014; Liu et al., 2018). However, if we have narrow-sense heritability estimates before constructing an RVM model, we are able to anticipate behaviour of the predictor, due to the higher weight of additive effects (as most genetic variance in populations is additive (Forsberg et al., 2017)).

### 3.1.3 *Comparison with related work*

Grinberg et al. (2018) recently compared several learning methods including forward stepwise regression, ridge regression, lasso regression, random forest, GBM, and Gaussian kernel SVM with two classical statistical genetics methods (BLUP and a linkage analysis done by Bloom et al. (2013)). Grinberg et al. used the coefficient of determination (*R* ^2^) as accuracy measure, and evaluated their models with one run of 10-fold cross validation. In Table 3, the columns “G: Best of Others” and “G: SVM” refer to Grinberg et al.‘s results. Also, the *R* ^2^ value in the RVM column belongs to the best RVM given in Table 1. Compared to the SVM, RVM models show better predictions overall. The Friedman and Nemenyi tests indicate that the differences mean of coefficients of determination for SVM and the different RVMs from Table 1 are all significant, with the exception of SVM compared to Gaussian RVM (*p* = 1*e* − 21 for the Friedman test, *p*-values for Nemenyi test are given in Table 2).

**TABLE 2:** Nemenyi test *p*-values for SVM and RVMs.

	**SVM**	**Gaussian RVM**	**Linear Basis RVM**	**Linear RVM**
SVM	1.000000	0.128475	0.001	0.007769
Gaussian RVM	0.128475	1.000000	0.001	0.001000
Linear Basis RVM	0.001000	0.001000	1.000	0.001000
Linear RVM	0.007769	0.001000	0.001	1.000000

**TABLE 3:** RVM results versus Grinberg et al.‘s (G) (Grinberg et al., 2018). RVM represents the best performing RvM model and its standard deviation.

**Trait**	**G: Best of Others**	**G: SVM**	**RVM(std)**
Cadmium Chloride	GBM:0.797	0.565	0.639 (0.005)
Caffeine	GBM:0.250	0.234	0.233 (0.006)
Calcium Chloride	BLUP: 0.268	0.261	0.287 (0.007)
Cisplatin	GBM: 0.338	0.272	0.29 (0.005)
Cobalt Chloride	GBM: 0.460	0.448	0.466 (0.006)
Congo red	Lasso:0.504	0.487	0.491 (0.006)
Copper	GBM:0.452	0.338	0.379 (0.01)
Cycloheximide	SVM:0.529	0.529	0.514 (0.005)
Diamide	BLUP:0.498	0.486	0.483 (0.005)
E6 Berbamine	GBM:0.412	0.390	0.414 (0.008)
Ethanol	GBM:0.518	0.455	0.476 (0.006)
Formamide	GBM:0.350	0.240	0.25 (0.006)
Galactose	GBM:0.235	0.217	0.241 (0.008)
Hydrogen peroxide	SVM:0.399	0.399	0.397 (0.01)
Hydroquinone	BLUP:0.225	0.188	0.208 (0.009)
Hydroxyurea	GBM:0.337	0.301	0.342 (0.01)
Indoleacetic Acid	Bloom et al.:0.480	0.3	0.313 (0.007)
Lactate	Lasso:0.568	0.557	0.555 (0.005)
Lactose	GBM:0.582	0.565	0.574 (0.008)
Lithium Chloride	GBM:0.711	0.680	0.678 (0.006)
Magnesium Chloride	Bloom et al.:0.278	0.267	0.255 (0.005)
Magnesium Sulfate	Bloom et al.:0.519	0.378	0.41 (0.005)
Maltose	GBM:0.809	0.522	0.523 (0.005)
Mannose	GBM:0.255	0.215	0.213 (0.007)
Menadione	GBM:0.432	0.402	0.411 (0.006)
Neomycin	Lasso:0.614	0.597	0.596 (0.003)
Paraquat	Lasso:0.496	0.479	0.454 (0.005)
Raffinose	GBM:0.383	0.364	0.388 (0.007)
SDS	Lasso:0.411	0.383	0.398 (0.004)
Sorbitol	Bloom et al.:0.424	0.318	0.364 (0.009)
Trehalose	GBM:0.515	0.477	0.503 (0.005)
Tunicamycin	SVM:0.634	0.634	0.622 (0.006)
4-Hydroxybenzaldehyde	GBM:0.397	0.36	0.367 (0.008)
4NQO	GBM:0.636	0.542	0.512 (0.005)
5-Fluorocytosine	GBM:0.399	0.364	0.378 (0.008)
5-Fluorouracil	Lasso:0.552	0.546	0.559 (0.005)
6-Azauracil	GBM:0.315	0.279	0.304 (0.005)
Xylose	GBM:0.516	0.460	0.477 (0.004)
YNB	GBM:0.543	0.525	0.515 (0.009)
YNB:ph3	BLUP:0.195	0.166	0.177 (0.005)
YNB:ph8	BLUP:0.356	0.334	0.361 (0.006)
YPD	GBM:0.556	0.524	0.511 (0.008)
YPD:15C	Bloom et al.:0.432	0.333	0.356 (0.006)
YPD:37C	Bloom et al.:0.711	0.603	0.611 (0.006)
YPD:4C	GBM:0.485	0.421	0.438 (0.005)
Zeocin	GBM:0.495	0.475	0.472 (0.004)

The RVM is comparable to the best of the methods tested by Grinberg et al., except in six traits including Cadmium Chloride, Indoleacetic Acid, Magnesium Sulfate, Maltose, 4NQO, and YPD:37C in which GBM or Bloom et al.‘s method showed superiority. However, the mean broad sense heritability of these six traits is 0.88, and the mean narrow sense heritability is 0.66. This confirms that nonlinear techniques, including GBM and RVM, are competitive for predictions involving traits with high broad sense heritability. Also, we should note that we do not know about the stability of the methods experimented by Grinberg et al., as they ran only one 10-fold cross-validation, while the RVM shows high stability, as its standard deviations in 10 runs of 10-fold cross-validation were small.

### 3.2 Identifying influential markers

For identifying the most influential markers (SNPs) on the traits, we used our RVM ensemble architecture for ranking markers. An ensemble for a trait was composed of 400 linear basis RVMs, each with subsampling 50–60% of training data. As we are only interested in a small set of top ranked markers, we observed that the size of subsampling does not affect the results (data not shown). To demonstrate how well the ensemble RVMs act in identifying influential markers, we present the top ranked markers in three conditions (traits): Cadmium Chloride, Lithium Chloride, and Mannose. We chose Cadmium Chloride and Mannose as samples which the linear basis RVM showed excellent and poor phenotypic prediction accuracies (Table 1), respectively, while we chose Lithium Chloride for comparison to the work of Bloom et al. (2013). Also, these conditions are across a wide range of broad sense heritability: the broad sense heritability of Cadmium Chloride is 0.98, Mannose is 0.42 and Lithium Chloride is 0.87.

The ensemble RVMs for each of the three traits ranked around 90% of the markers with rank values in the range [1, 400]. The unranked markers indicate the markers that do not have any effect (even minor) on a trait. We define the most influential markers as those that are chosen by half of the RVMs in the ensemble as RVs, so in this dataset we will have less than ten influential markers in the three traits. The ranked markers indicate those who may have positive or negative effects on a trait. In other words, we not only find the markers which have additive effects on yeast growth in an environment, but also we find those which have adverse effects on growth.

#### 3.2.1 *Comparison with related work*

Previously, Bloom et al. (2013) conducted a linkage analysis with high statistical power to map functional QTL in all 46 traits. They found that nearly the entire additive genetic contribution to heritable variation (narrow-sense heritability) in yeast can be explained by the detected loci. Bloom et al. specifically showed that for one trait (Lithium Chloride), the loci detected by their method explained most of the heritability.

**FIGURE 2:**
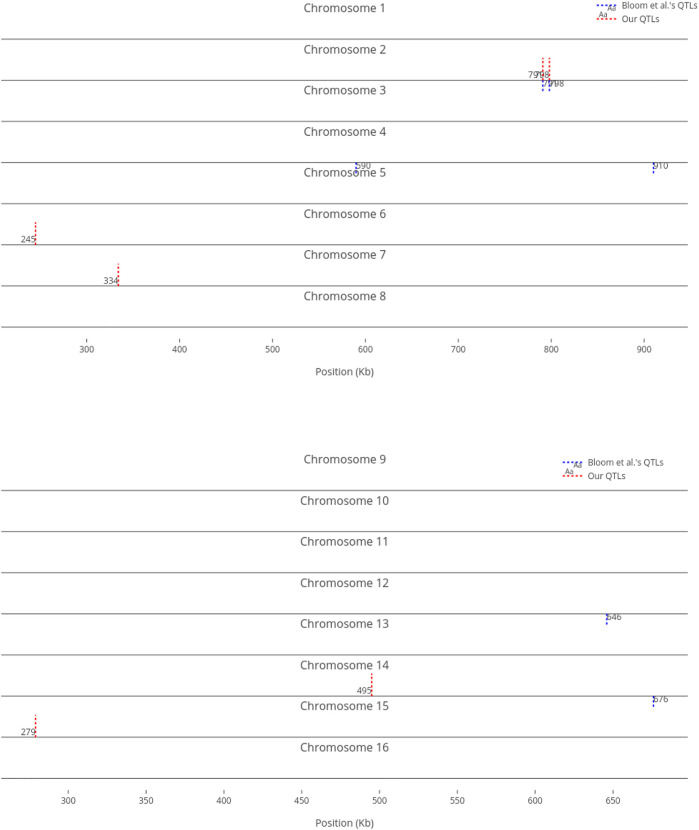
Top six influential markers on growth in Cadmium Chloride recognized by ensemble RVMs versus Bloom et al.‘s six QTL.

**FIGURE 3:**
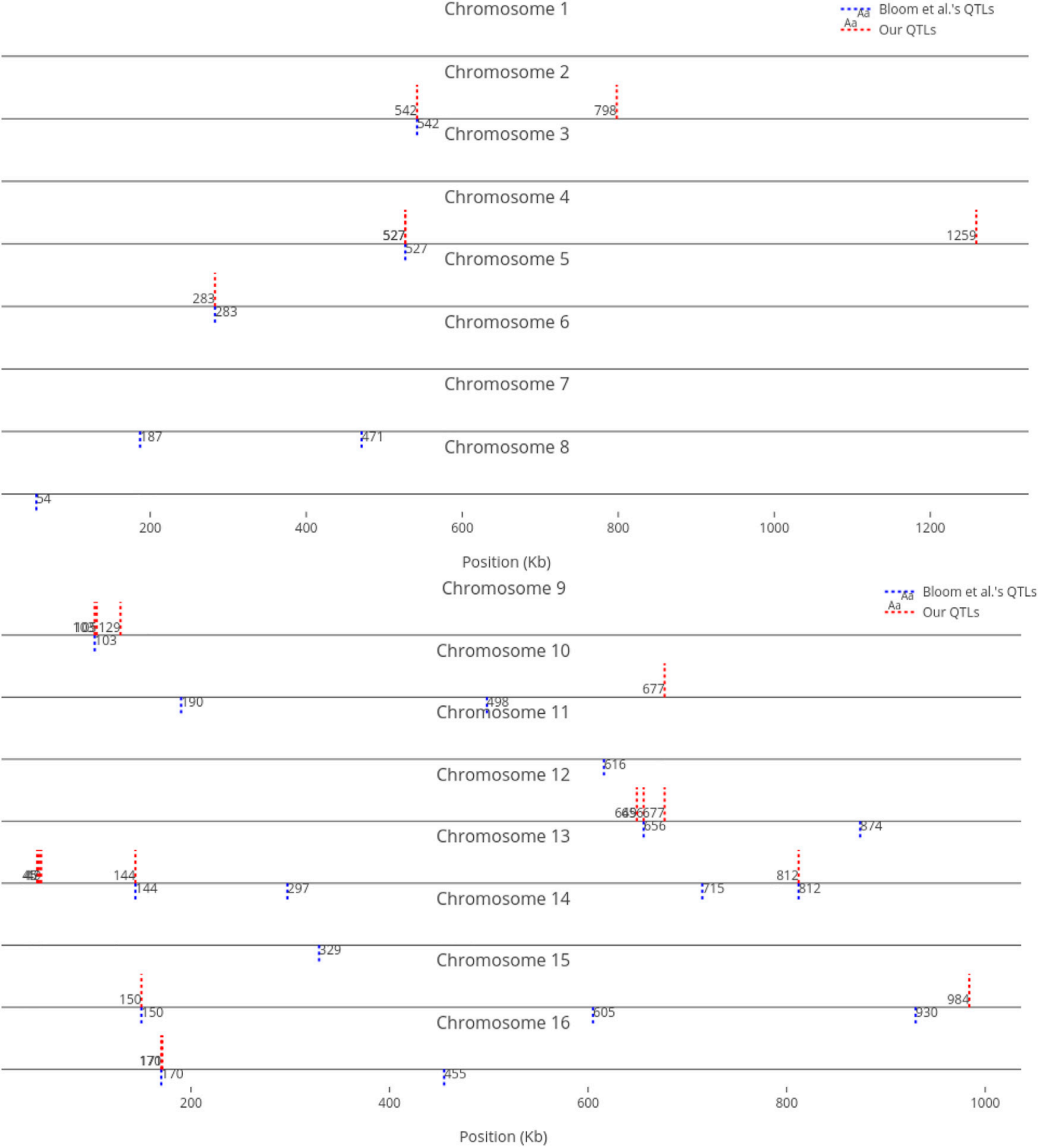
Top 22 influential markers on growth in Lithium Chloride recognized by ensemble RVMs versus Bloom et al.‘s 22 QTL. (To view the improved version of (b) follow the link:(b) - Interactive Chart).

**FIGURE 4:**
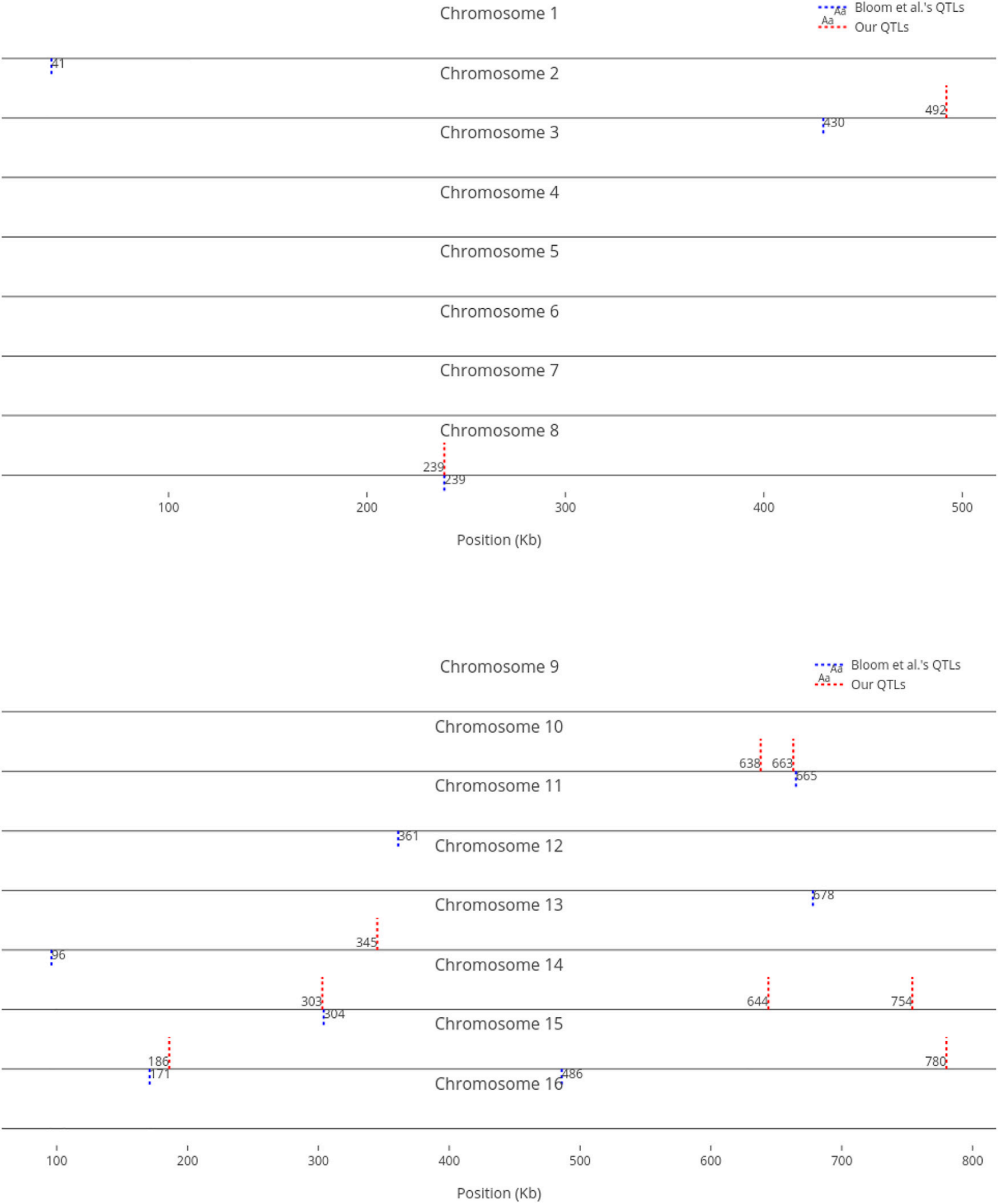
Top 10 influential markers on growth in Mannose recognized by ensemble RVMs versus Bloom et al.‘s 10 QTL.

We compare our identified influential markers in three traits to Bloom et al.‘s QTL. Bloom et al. found 6, 22, and 10 additive QTL in Cadmium Chloride, Lithium Chloride, and Mannose, respectively. Therefore, we chose the top 6, 22, 10 ranked SNPs in the three traits as well. Figures 2–4 show results in each of the three traits accordingly. Each of the figures includes two parts (a) and (b) corresponding to the map of yeast chromosomes 1-8 and 9–16, respectively. The results were demonstrate that the markers identified by the RVM ensembles have similar distribution to the Bloom et al.‘s QTL. Also, the RVM ensembles were relatively successful in finding the exact markers in the traits (33% match rate in Cadmium Chloride, 36% in Lithium Chloride, and 40% in Mannose). We note that the highest match rate among the three traits belongs to Mannose in which the linear basis RVM had poor prediction accuracy. This could be an advantage of the RVM being capable of recognizing true “representatives” of a population, despite unacceptable predictions. Another advantage is in the ranking system, where we can always recognize the effect of a marker on a trait with its weight, even in the small set of top-ranked markers. However, we can also go further and conclude that those top ranked markers that are close to each other (e.g., markers at loci 649 kb, 656 kb, and 677 kb on Chromosome 12 in Figure 3) suggest to a higher impact of a locus near to those markers on a trait due to genetic linkage.

**FIGURE 5:**
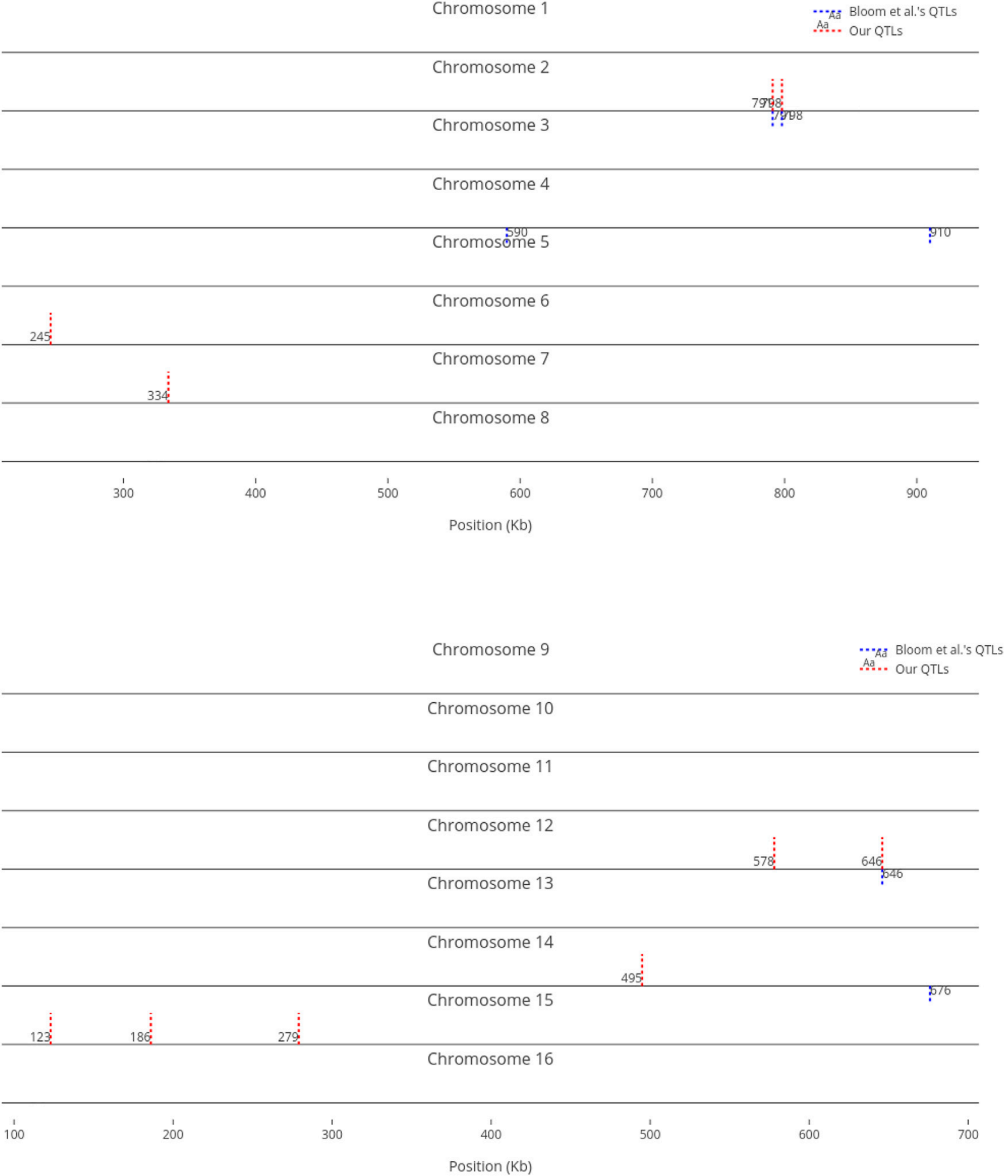
Top 10 influential markers on growth in Cadmium Chloride recognized by ensemble RVMs versus Bloom et al.‘s six QTL.

For comparison purposes, we only provided an equal number of top ranked markers to Bloom et al.‘s QTL. However, when we decrease the threshold, the number of influential markers would increase. For instance, Figure 5 shows the top ten (instead of six) most influential markers in Cadmium Chloride. In this case, another additive QTL in chromosome 12 is identified (i.e., at position 464 kb). As not all influential markers have additive effects, the identified markers which are distant from Bloom et al.‘s QTL present a good set of candidates for further investigation, to determine if they have non-additive effects with other loci.

## 4 Conclusion

In this research, we studied how RVMs perform on growth prediction of yeast in 46 different environments, comparing its performance with other learning methods such as SVM and GBM. Our obtained phenotype prediction accuracies suggest that RVM shows positive results, and can be used as an alternative method in genomic selection. It is well-known that no machine learning technique performs best in all datasets (Ogutu et al., 2011; Grinberg et al., 2018).

We investigated different kernels in RVM. We illustrated how different linear RVMs, i. e, linear kernel RVM and linear basis RVM, perform in phenotype prediction. We observed that Gaussian RVMs had the best accuracies.

We also investigated the relationship between different heritability measures and RVM prediction accuracies. The results indicate an strong association between narrow-sense heritability and prediction accuracy in RVMs. On the other hand, new research points out that the most genetic variance in populations is additive (Forsberg et al., 2017). Therefore, if the heritability is known in advance, we can consequently anticipate the performance of the model before constructing it.

The last part of the experiments was devoted to identifying most influential markers on the traits, as well as non-relevant markers. We chose three traits with different phenotype prediction accuracies as samples, and demonstrated how well our RVM ensembles work to rank the markers in each trait, comparing the results with other research which used a traditional linkage analysis to find additive QTL. The comparison validates the results of RVM ensembles in finding markers with additive effects. However, we can learn more from the RVM ensembles, as those are capable of identifying both growth-increasing and growth-decreasing markers in yeast.

It may be observed that our ensemble linear basis RVM for feature selection takes in to account only linear relationships. Although this linear separability is a reasonable assumption for high dimensional data, it is desirable to investigate nonlinear basis substitution, particularly Gaussian functions, to handle nonlinear relationships. Gaussian basis RVM still gives feature RVs as each Gaussian basis in the model operates on a different dimension (feature). However, employing Gaussian basis RVM requires setting not only the variance (*σ*_*m*_) in each Gaussian basis function in (1), but also the mean or center (*μ*_*m*_): $\rho_{m}\left(x\right)=\exp\left(-{\left(x^{\left[m\right]}-\mu_{m}\right)}^{2}/{\sigma_{m}}^{2}\right)$ , where **x** ^[*m*]^ refers to the *m*th feature in an input vector **x** with *M* dimensions. Investigating any appropriate approach, with acceptable computational complexity, for choosing parameters in Gaussian basis RVMs, and employing these RVMs in an application remain as future work.

## Data Availability

Publicly available datasets were analyzed in this study. This data can be found here: http://genomics-pubs.princeton.edu/YeastCross_BYxRM/data.shtml.
